# Clinical outcomes of patients with diffuse large B-cell lymphoma and concomitant rheumatoid arthritis: a nationwide Danish register-based cohort study

**DOI:** 10.1016/j.ero.2025.11.009

**Published:** 2025-12-06

**Authors:** Bergur Magnussen, Lene Wohlfart Dreyer, Salome Kristensen, Tarec Christoffer El-Galaly, Lasse Hjort Jakobsen, Lene Mellemkjær, Mikkel Simonsen, Peter Brown, Rasmus Westermann

**Affiliations:** 1 Center of Rheumatic Research Aalborg (CERRA), Aalborg University Hospital, Aalborg, Denmark; 2 Clinical Cancer Research Center, Aalborg University Hospital, Aalborg, Denmark; 3 The DANBIO register, Copenhagen, Denmark; 4 Department of Hematology, Aarhus University Hospital, Aarhus, Denmark; 5 Department of Clinical Epidemiology, Aarhus University Hospital, Aarhus, Denmark; 6 Department of Molecular Medicine, Aarhus University Hospital, Aarhus, Denmark; 7 Department of Clinical Medicine, Aarhus University, Aarhus, Denmark; 8 Department of Haematology, Clinical Cancer Research Unit, Aalborg University Hospital, Aalborg, Denmark; 9 Danish Cancer Institute, Copenhagen, Denmark; 10 Department of Mathematical Sciences, Aalborg University, Aalborg, Denmark; 11 Department of Hematology, Copenhagen University Hospital Rigshospitalet, Copenhagen, Denmark

## Abstract

**Objectives:**

This register-based cohort study aimed to investigate clinical cancer outcomes in patients with rheumatoid arthritis (RA) who develop diffuse large B-cell lymphoma (DLBCL) compared with those in patients without RA but with DLBCL.

**Methods:**

The study included all patients with RA and DLBCL aged ≥18 years in the Danish National Lymphoma Registry who received first-line treatment between January 1, 2006, and May 21, 2022. RA diagnoses (exposure) and patients without RA (comparator) at the time of DLBCL diagnosis were identified through linkage with the Danish Rheumatology Quality Register and the Danish National Patient Registry. Patients were followed up from DLBCL treatment initiation until emigration, death, outcomes of interest, or May 21, 2022. Hazard ratios (HRs) and 95% CIs were estimated for outcomes including overall survival (OS), progression-free survival (PFS), and hospital admissions.

**Results:**

The final study cohort included 136 patients with RA and 5372 without RA. Adjusted HRs for OS and PFS in patients with RA were 1.02 (95% CI, 0.74-1.39) and 0.86 (95% CI, 0.65-1.14), respectively, compared with those of patients without RA. Two-year OS was 81.9% in patients with RA and 77.8% in those without RA, with 2-year PFS of 72.6% and 72.4%, respectively. For other outcomes, HRs were <1 for patients with RA, with a statistically significant reduction in hospital admissions: 0.56 (95% CI, 0.44-0.72) in adjusted calculations.

**Conclusions:**

In conclusion, patients with RA and DLBCL did not experience worse survival outcomes or increased hospitalisation rate during therapy.

**WHAT IS ALREADY KNOWN ON THIS TOPIC**

• Current studies that have investigated the clinical outcomes of patients with rheumatoid arthritis (RA) and diffuse large B-cell lymphoma (DLBCL) show conflicting results, with some studies indicating worse outcomes and others showing better. Additionally, most current studies only investigate overall survival while neglecting other important outcomes such as progressive-free survival, remission rate, and hospital admission.

**WHAT THIS STUDY ADDS**

• In our study, the patients with RA did not show worse outcomes, and in fact, when looking at hospital admissions, patients who had RA and DLBCL had significantly fewer admissions compared with patients without RA.

**HOW THIS STUDY MIGHT AFFECT RESEARCH, PRACTICE OR POLICY**

• Our study may contribute to the literature, suggesting that DLBCL that develops in an RA population may differ in some way from DLBCL that develops in the general population. It may also offer reassurance to patients with RA who are diagnosed with DLBCL.

## INTRODUCTION

Diffuse large B-cell lymphoma (DLBCL) is the most common subtype of non-Hodgkin lymphoma (NHL) worldwide [1]. It is well established that the risk of NHL is elevated in patients with various inflammatory rheumatic diseases (IRDs) [[2], [3], [4], [5]] including rheumatoid arthritis (RA) [4,5].

Current literature suggests that patients with RA have, on average, a 2-fold increased risk of developing lymphoma compared with the general population [[6], [7], [8], [9], [10]]. Notably, the proportion of DLBCL among lymphomas in RA patients appears to be disproportionately high. One study found that 67% of lymphomas in RA patients were of the DLBCL subtype, compared with 30% to 40% in the general population [6]. One possible explanation for the increased DLBCL risk in patients with RA is the RA-associated chronic inflammation with stimulation of B-cells [9]. It has also been shown that the risk of developing lymphoma is highly associated with the severity of RA disease activity [11]. Although there has been considerable research into the risk of DLBCL in patients with RA [6,12,13], studies that examine DLBCL outcomes in patients with RA are very heterogeneous in design and limited in the number of included patients. Given that RA patients are known to experience immune dysregulation, fully understanding how RA influences DLBCL outcomes in terms of survival and complication rates would be of value for the patients and clinicians [14]. Existing studies on the clinical outcomes of patients with DLBCL and RA have shown mixed results. The largest study was by Ji et al [15], which included 329 patients with RA and 56,936 without RA, and it found that patients with NHL and RA had a higher 1-year mortality reporting a hazard ratio (HR) of 1.33 (95% CI, 1.26; 1.59) compared with patients without RA. However, this Swedish study considered all NHLs collectively rather than focusing specifically on DLBCL, and their study period included DLBCL patients far back (1961-2006). Other studies found no differences in lymphoma outcomes in patients with RA versus patients without RA [[16], [17], [18]]. General limitations of previously conducted studies of DLBCL outcomes in patients with RA include small sample sizes with limited power to detect minimally clinically important differences and reporting of outcomes in groups of patients with various types of NHL without a specific focus on DLBCL [15,17,18]. Those studies also do not account for other important clinical outcomes related to the risk of treatment complications in patients with lymphoma and RA versus patients without RA. We therefore aimed to investigate whether patients with DLBCL and RA experienced worse clinical outcomes compared with DLBCL patients without RA.

## METHODS

### Study design

We performed an observational cohort study on Danish patients with DLBCL who were diagnosed between January 2006 and 21 May 2022 and compared various clinical outcomes of patients with concomitant RA with those without RA.

### Data sources

In Denmark, individual-level data can be linked across various registries through a unique civil registration number (CPR-number) registered in the Danish Civil Registration System (CRS) [19,20]. Via CRS, we were also able to collect information on date of birth, biological sex, migration status, and vital status. DLBCL diagnoses and DLBCL-specific clinicopathological features were extracted from The Danish National Lymphoma Registry (LYFO). LYFO is a Danish registry that became nationwide in 2000 with the purpose of monitoring lymphoma treatment quality and outcomes in Denmark. LYFO has a completeness between 98.1% and 100% and a positive predictive value (PPV) ranging from 93.4% to 100% [21,22]. From LYFO, we obtained data on DLBCL diagnoses, Ann Arbor stages, presence of B-symptoms, PS, bulky disease, blood levels of lactate dehydrogenase (LDH), and remission and relapse status. Information on relapse status was also taken from the Danish pathology register (DPR). Since 1999, the DPR has been updated daily with patient, specimen, workload data, and requisition numbers. Diagnoses are coded using the mandatory Danish Systematized Nomenclature of Medicine [23]. The Danish Rheumatology Quality Register (DANBIO) was used to identify patients with RA. All patients who have received treatment with biological disease-modifying antirheumatic drugs (bDMARDs) have been registered in DANBIO since 2000, and since 2006, all RA patients regardless of treatment have been registered. The validity of RA diagnoses in DANBIO has been reported as high with a PPV of 96% and a completeness of 91% [[24], [25], [26]]. The Danish patient registry (DNPR) is one of the world’s first nationwide hospital registries and contains information on all inpatients at Danish hospitals since 1977 and outpatients since 1995. Diagnoses in the DNPR are classified according to the International Classification of Diseases 10th Edition (ICD-10) [27,28]. In DNPR, all hospital admissions and their durations are listed, including admissions to intensive care units (ICUs) that can be found using procedure codes NABB (intensive treatment) and NABE (intensive observation) [29]. All non-RA IRD diagnoses were identified in DNPR using ICD-10 codes, see Supplementary material S1. For the list of ICD-10 codes defining hospital admission related to infections, see Supplementary material S2.

### Study population

The primary cohort was identified in LYFO and consisted of all patients aged 18 years or more with a registered DLBCL diagnosis between 1 January 2006 and 21 May 2022 who had been treated with first-line rituximab, cyclophosphamide, doxorubicin, vincristine, and prednisone (R-CHOP) or other equally effective treatments. For the full list of included treatments, see Table 1.

**Table 1:** Baseline characteristics and clinicopathological features of DLBCL patients

Group	DLBCL + RA	DLBCL without RA
Number of patients	N = 136	N = 5372
**Sex**		
Female	84 (61.8%)	2237 (41.6%)
Male	52 (38.2%)	3135 (58.4%)
**Age at start of chemotherapy**		
Median (IQR), y	72.3 [65.5,77.9]	68.6 [59.0,76.1]
**Calendar year of DLBCL**		
2006-2010	22 (16.2%)	1467 (27.3%)
2010-2015	50 (36.8%)	1721 (32.0%)
2015-2022	64 (47.1%)	2184 (40.6%)
**Performance status**		
0-1	>110^a^(≈80%)	4462 (83.1%)
2+	22 (16.2%)	895 17.8%)
Missing	<3^a^	15 (0.3%)
**Ann Arbor stage**		
I-II	>30^a^(≈20%)	1901 (35.4%)
III-IV	101 (74.3%)	3417 (63.6%)
Missing	<3^a^	54 (1.0%)
**Extra nodal involvement**		
0-1	90 (66.2%)	3740 (69.6%)
>2	46 (33.8%)	1632 (30.4%)
**Bulky disease**		
≤10cm	114 (83.8%)	4022 (74.9%)
>10	17 (12.5%)	1147 (21.4%)
Missing	5 (3.7%)	203 (3.8%)
**Presence of B-symptoms**		
Yes	55 (40.4%)	2218 (41.3%)
No	>75 (≈55%)^a^	3028 (56.4%)
Missing	<3^a^	126 (2.3%)
**IPI index**		
Low	28 (20.6%)	1472 (27.4%)
Intermediate	68 (50.0%)	2616 (48.7%)
High	35 (25.7%)	1101 (20.6%)
Missing	5 (4.1%)	183 (3.4%)
**Blood levels of LDH**		
Elevated	76 (55.9%)	2903 (54.0%)
Not elevated	57 (41.9%)	2351 (43.8%)
Missing	3 (2.2%)	118 (2.2%)
**Chemotherapy**		
R-CHOP	121 (89.0%)	4647 (86.5%)
R-CHOEP	8 (5.9%)	562 (10.5%)
R-CEOP	4 (2.2%)	108 (2.0%)
R-CVP	3 (2.9%)	55 (1.0%)
**Number of chemo cycles**		
≤6	121 (89.0%)	4814 (89.6%)
>6	15 (11.0%)	558 (10.4%)
**Received bDMARD treatment**		
Any	42 (30.9%)	0
Rituximab	<3	0
**Sjögren’s disease**		
Yes	8 (5.9%)	0
No	128 (94.1%)	0

bDMARD, biological disease-modifying antirheumatic drugs; DLBCL, diffuse large B-cell lymphoma; DLBCL + RA, diffuse large B-cell lymphoma and rheumatoid arthritis; DLBCL without RA, diffuse large B-cell lymphoma and no RA or any other inflammatory rheumatic disease; IPI index, international prognostic index; LDH, lactate dehydrogenase; RA, rheumatoid arthritis; R-CHOEP, R-CHOP+etoposid; R-CHOP, rituximab, cyclophosphamide, doxorubicin, vinocristine, prednisone; R-CVP, R-CP+vincristine sulfate.

a Estimates of percentages and not numbers are shown due to Danish law concerning indirect anonymisation.

### Exposure and comparators

By linking data from LYFO and DANBIO, we identified all patients registered with a diagnosis of RA in DANBIO and a diagnosis of DLBCL in LYFO. Patients were included in the exposure group (DLBCL + RA) if their RA diagnosis preceded their DLBCL diagnosis. For the comparator group (DLBCL without RA), we included all patients with a DLBCL diagnosis recorded in LYFO and no prior diagnosis of an RA or any other IRDs, as collected from the DNPR.

### Outcomes and follow-up

The following clinical outcomes were investigated: overall survival (OS), progression-free survival (PFS), first hospital admission, first admission related to infections or at ICUs, number of inpatient bed days, and ICU admission days. All patients were followed from the date of DLBCL treatment start until the first of the following events: **outcomes of interest,** emigration, death, or 21 May 2022. PFS was calculated as the time from the diagnosis of DLBCL until relapse/progression or death from any cause. For the endpoints, hospital admission and admission to an ICU specifically, follow-up commenced on the start of the first chemotherapy session and was terminated 3 months after the last chemotherapy session or at the occurrence of each respective event: first admission to a hospital, or at first admission to ICUs. This follow-up period was chosen to try and capture only events likely related to the patients’ DLBCL diagnosis and treatment.

### Statistical methods

Median follow-up was calculated using the reverse Kaplan-Meier method. For all time-to-first event outcomes, the Cox proportional hazard model was used to calculate HRs and 95% CIs. For the total number of inpatient bed days and ICU admission days, incidence rate ratios (IRRs) and 95% CI were estimated by Poisson regression, with RA status as the explanatory variable and the logarithm of person-time as an offset [30]. All calculations were performed unadjusted and adjusted for age, sex, calendar year of DLBCL diagnosis, PS, Ann Arbor stage, tumour bulk, and elevated/normal blood levels of LDH. For OS and PFS, survival curves with 95% **CI** were computed using the Kaplan-Meier estimator. For hospital admissions, where death was considered a competing event, the cumulative incidence was estimated using Aalen-Johansen cumulative incidence curves. All analyses were performed in R (version 4.2.2). Survival functions, cumulative incidence curves, and HR were calculated using the **‘Survival’** and **‘Survminer’ packages. The ‘Cobs’ package was used to smooth all survival curves** due to Danish legislation concerning patient anonymity. The ‘Glm’ package was used for Poisson regression curves, IRRs, and 95% CI.

### Sensitivity analysis

In a sensitivity analysis, we looked at hospital admissions after removal of all admissions with a procedure code for BOHJ11 (treatment with rituximab) and BWHA (treatment with cystostatics). The intent was to avoid admissions that were solely related to the administration of R-CHOP-like treatment.

### Subgroup analysis

Three subgroup analyses were performed. In the first, the DLBCL + RA group was stratified by (1) RA patients previously treated with any form of bDMARDs, ie, tumour necrosis factor inhibitors, interleukin 6 inhibitors, CD80/CD86 costimulation inhibitors, and CD20 inhibitors, or (2) RA patients who had never received treatment with any form of bDMARD. This was done because patients treated with bDMARDs are expected to have more severe RA disease activity, which is a known risk factor for cancer development. Therefore, comparing the DLBCL clinical outcomes of these 2 groups of RA patients could offer some insight into the relationship between RA disease activity and DLBCL outcomes. In the second subgroup analysis, we stratified the DLBCL + RA group by seropositivity using ICD-10 codes M05.9 (seropositive) and M06.9 (seronegative). Seropositive RA patients often differ from seronegative RA patients regarding prognosis and other outcomes [[31], [32], [33]]. For the final subgroup analysis, we stratified the RA cohort based on recent methotrexate use. Recent methotrexate use was defined as having a recorded methotrexate treatment within 6 months prior to the DLBCL diagnosis. This analysis was conducted because there have been reports of methotrexate induced lymphoproliferative disorders, which often regress spontaneously after discontinuation of the drug [[34], [35], [36], [37]].

## RESULTS

A total of 7263 patients diagnosed with DLBCL were registered in LYFO. Among them, 455 patients had a registered diagnosis of RA or other IRDs before developing DLBCL, comprising the DLBCL without RA group. We also identified 166 patients with both DLBCL and an RA diagnosis in DANBIO. Of these, 136 (81.2%) received R-CHOP-like treatment. Among the 6808 DLBCL without RA patients, 5372 (80.0%) received R-CHOP-like treatment. Median follow-up for the 136 DLBCL + RA patients was 7.48 years (IQR 4.0 to 11.5) and 7.08 years (IQR 3.3 to 11.5) for the comparator group of 5372 DLBCL without RA patients. The median age at DLBCL diagnosis was 72.3 years (IQR 65.5 to 77.9) for DLBCL + RA and 68.6 years (IQR 59.0 to 76.1) for DLBCL without RA patients. Among DLBCL + RA patients, 61.8% were female, whereas among DLBCL without RA patients, 41.6% were female. A higher proportion of patients were diagnosed with Ann Arbor stage III-IV in the group of DLBCL + RA (74.3%) compared with DLBCL without RA (63.6%). A full list of baseline characteristics is shown in Table 1.

### Outcomes

**Figure 1:**
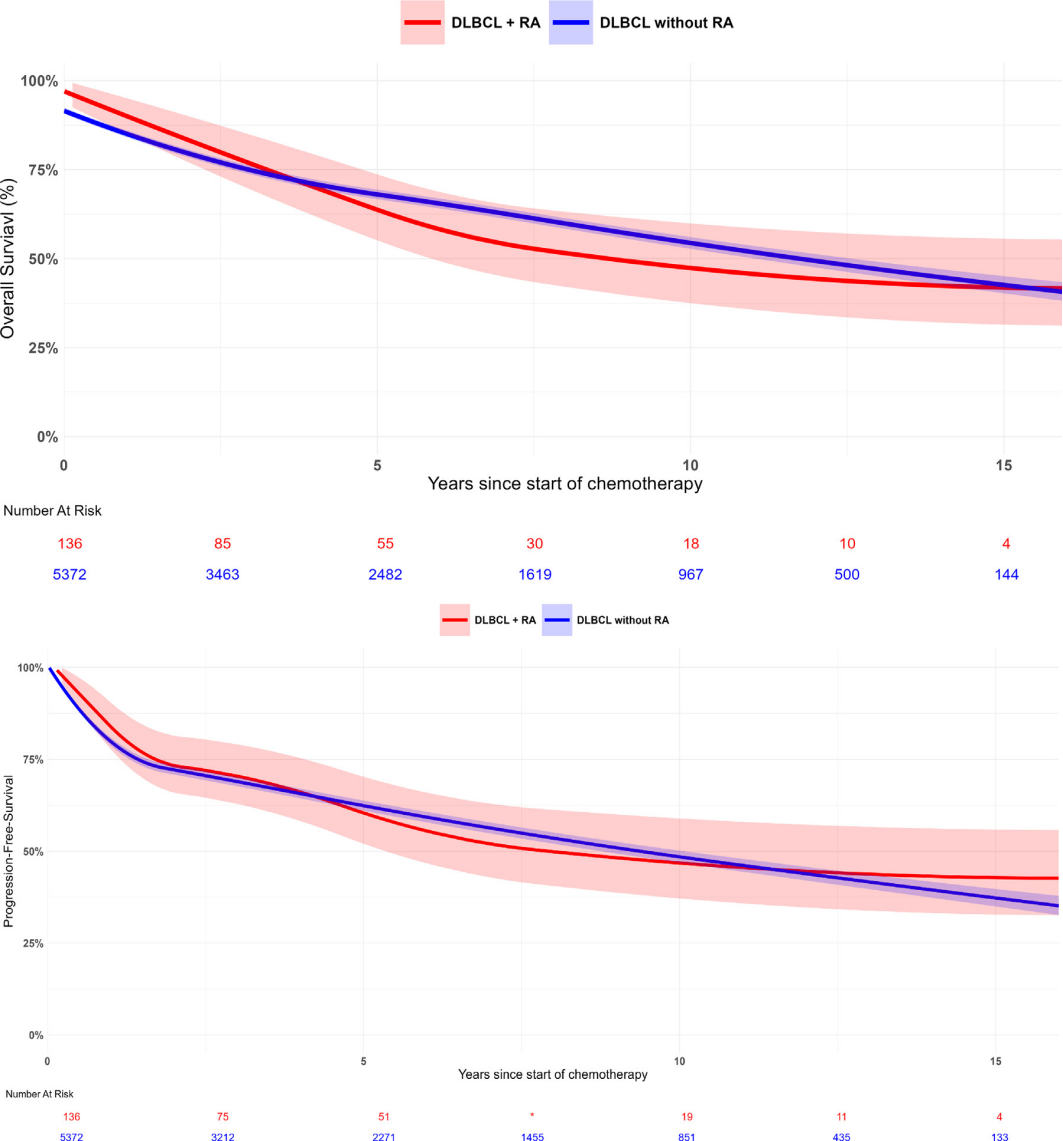
Smoothed Kaplan-Meier curves for overall survival (top) and progressive-free survival (bottom). *Number at risk censored due to Danish law concerning indirect anonymisation. DLBCL, diffuse large B-cell lymphoma; DLBCL + RA, diffuse large B-cell lymphoma and rheumatoid arthritis; DLBCL without RA, diffuse large B-cell lymphoma and no inflammatory rheumatic disease; RA, rheumatoid arthritis.

Crude OS at 2 years was 81.9% (95% CI, 75.5%; 88.8%) and 78.0% (95% CI, 76.9%; 79.1%) for DLBCL + RA and DLBCL without RA patients, respectively. See Figure 1*.* Unadjusted and adjusted HRs for OS were 1.06 (95% CI, 0.81; 1.40) and 1.01 (95% CI, 0.74; 1.39) with DLBCL without RA patients treated as reference. Two-year PFS was 72.6% (95% CI, 66.2%; 81.6%) and 72.4% (95% CI, 71.4%; 73.8%) for DLBCL + RA and DLBCL and DLBCL without RA patients, respectively. See Figure 1*.* The adjusted HR for PFS was 0.86 (95% CI, 0.65; 1.14) for patients with DLBCL + RA compared with DLBCL without RA patients.

DLBCL + RA patients had a lower incidence rate of 159 admission days per 100 person-years (PYs) compared with 245 admission days per 100 PYs for those with DLBCL without RA (Table 2). IRR for inpatient bed days was 0.73 (95% CI, 0.68; 0.79) for DLBCL + RA patients compared with DLBCL without RA patients.

**Table 2:** Primary analysis: overall survival, progression-free survival, complete remission, death at 3 months, inpatient bed days, and hazard ratios/incidence rate ratios (95% CI) for investigated outcomes comparing DLBCL + RA patients with DLBCL without RA patients

Group	DLBCL + RA	DLBCL without RA
Number of patients	N = 136	N = 5372
**Overall survival**		
2-y overall survival	81.9% (75.4; 88.9)	77.8% (76.7; 78.9)
5-y overall survival **Progressive-free survival**	63.2% (54.6; 73.2)	68.7% (67.6; 70.2)
2-y progression-free survival	72.6% (66.2; 81.6)	72.4% (71.4; 73.8)
5-y progression-free survival	58.8% (50.1; 68.8)	62.2% (61.1; 63.8)
**Complete remission (%)**	87.5%	81.4%
**Death at 3 mo (%)**	2.21%	5.2%
**Hospital admission**		
Total number of inpatient bed days	1091	71,737
Inpatient bed days per 100 person-years (IR)	159	245
Total number of admission days due to infection	228	11,329
Infection related to inpatient bed days 100 person-years (IR)	33	39
Total number of admission days in ICUs^a^	0	4398
**Crude HRs and IRRs**		
HR for death (95% CI)	1.06 (0.81; 1.40)	Reference
HR for progressive-free survival (95% CI)	0.97 (0.75; 1.26)	Reference
HR for first hospital admission (95% CI)	0.62 (0.50; 0.78)	Reference
HR for first serious infection (95% CI)	0.81 (0.53; 1.23)	Reference
IRR for inpatient bed days (95% CI)	0.82 (0.77; 0.87)	Reference
**Adjusted HRs and IRRs**		
HR for death^b^ (95% CI)	1.02 (0.74; 1.39)	Reference
HR for progression-free survival^b^ (95% CI)	0.86 (0.65; 1.14)	Reference
HR for hospital admission^b^ (95% CI)	0.56 (0.44; 0.72)	Reference
HR for serious infections^b^ (95% CI)	0.70 (0.44; 1.11)	Reference
IRR for inpatient bed days^b^ (95% CI)	0.73 (0.68; 0.79)	Reference

DLBCL + RA, diffuse large B-cell lymphoma and rheumatoid arthritis; DLBCL without RA, diffuse large B-cell lymphoma and no RA or any other inflammatory rheumatic disease; HR, hazard ratio; ICU, intensive care unit; IR, incidence rate; IRR, incidence rate ratio; LDH, lactate dehydrogenase.

a Due to no admissions to ICUs for the DLBCL + RA group, comparative analysis was unable to be performed, and 95% CI.

b Adjusted for sex, age at lymphoma, performance status, Ann Arbor stage, extranodal involvement, presence of B-symptoms, tumour bulk, and blood levels of LDH.

For first-time hospital admission, we found statistically significantly lower HRs in both the unadjusted and adjusted calculations, with an HR of 0.56 (95% CI, 0.44; 0.72) in the adjusted calculation. The full list of outcomes is shown in Table 2.

**Figure 2:**
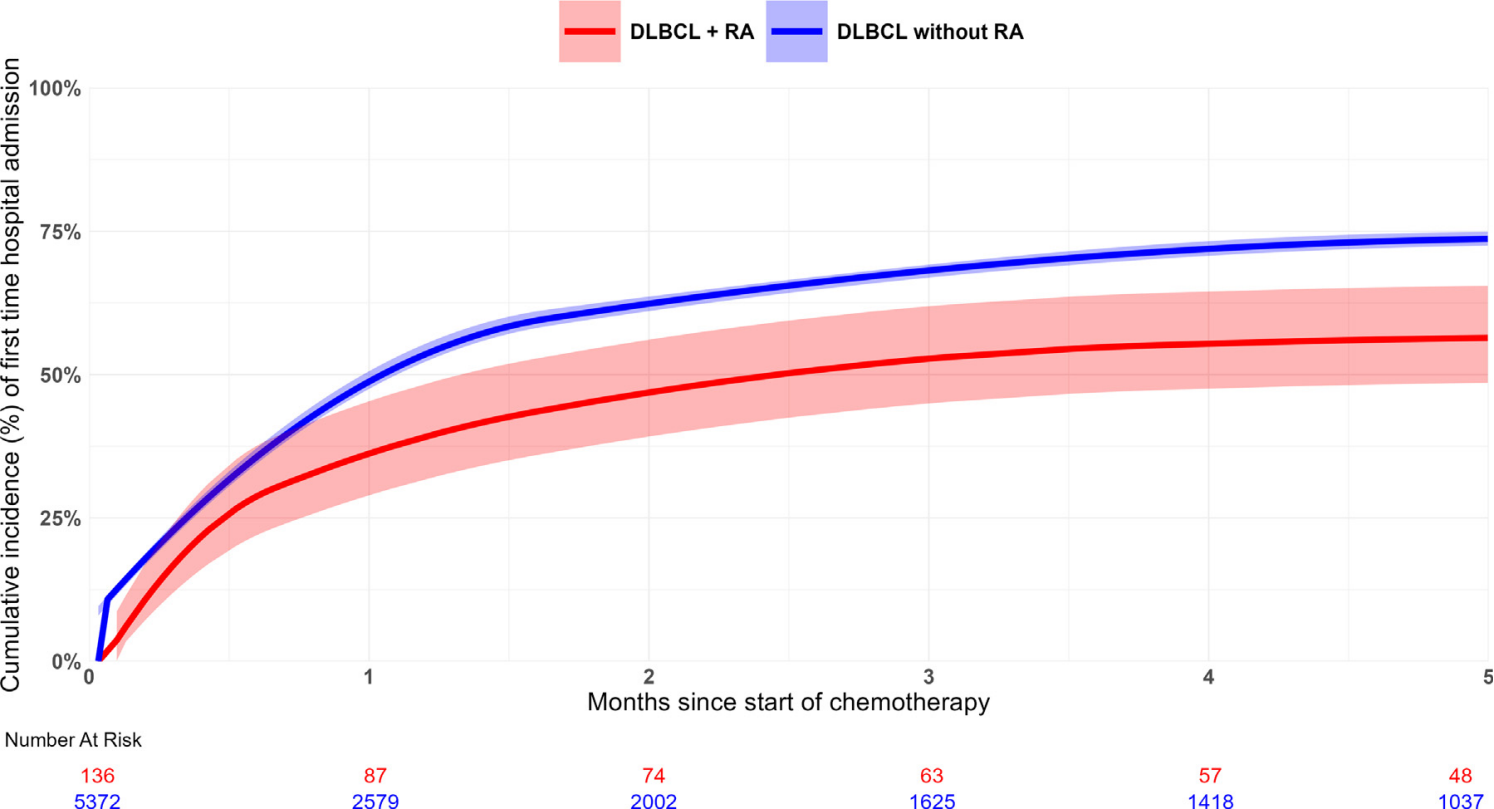
Cumulative incidence of hospital admission. DLBCL, diffuse large B-cell lymphoma; DLBCL + RA, diffuse large B-cell lymphoma and rheumatoid arthritis; DLBCL without RA, diffuse large B-cell lymphoma and no inflammatory rheumatic disease; RA, rheumatoid arthritis.

The 5-month cumulative risk of hospital admission was 56.1% for DLBCL + RA patients, compared with 73.5% for those with DLBCL without RA patients. See Figure 2.

### Sensitivity analysis for hospital admission

When excluding hospital admission related to administration of chemotherapy, the adjusted HR for hospital admission was very close to the primary analysis for DLBCL + RA patients compared with DLBCL without RA patients: HR, 0.56 (95% CI, 0.40; 78). In addition, the IRR for hospital admissions was also very close to the main analysis: IRR, 0.83 (95% CI, 0.78; 0.88).

### Subgroup analysis

**Figure 3:**
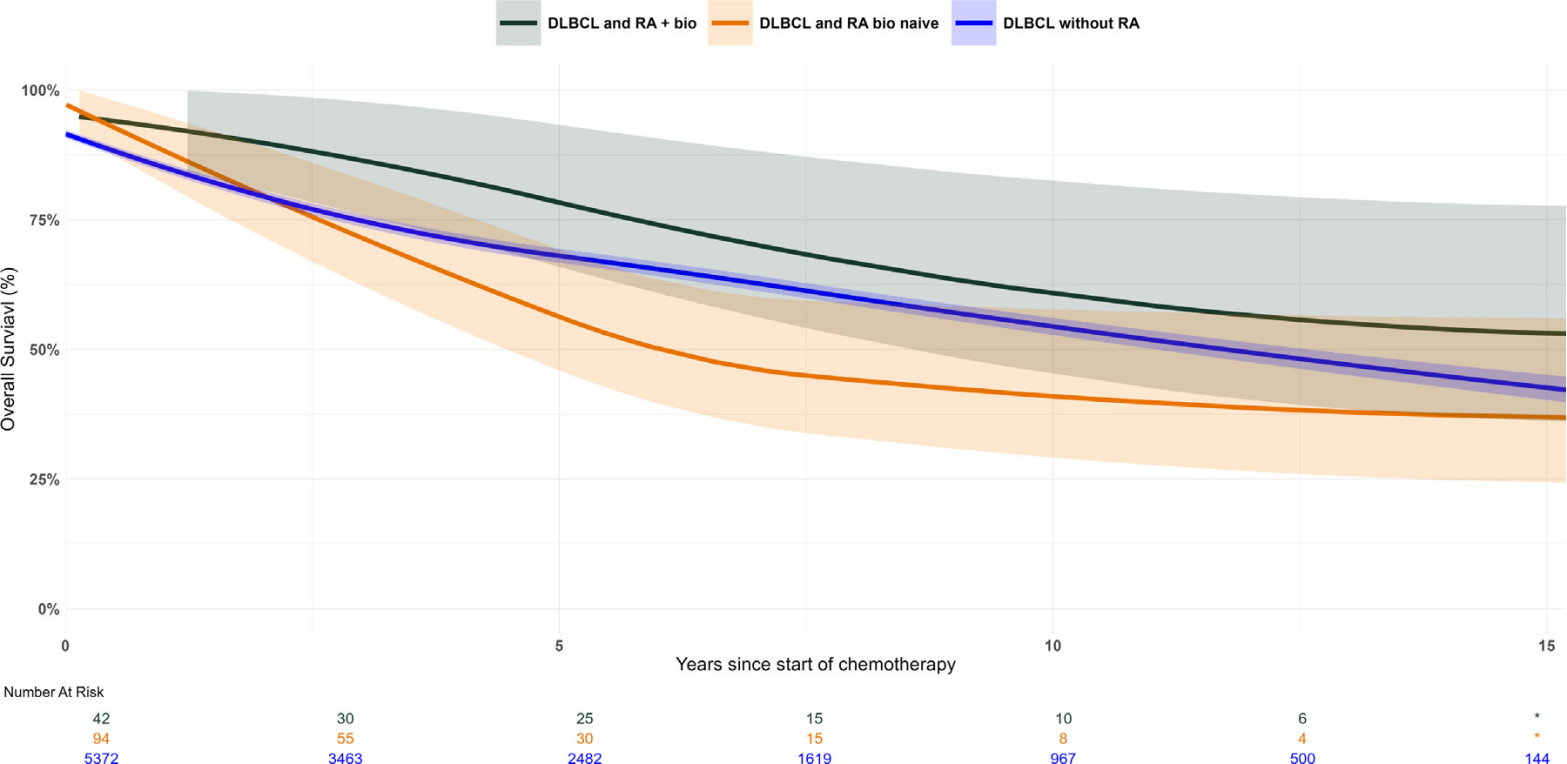
Smoothed curve for overall survival in subgroup analysis comparing patients with DLBCL and no IRDs with patients with DLBCL and seropositive RA, and DLBCL patients with seronegative RA. *Number at risk censored due to Danish law on indirect anonymisation. DLBCL without RA, diffuse large B-cell lymphoma and no inflammatory rheumatic disease; DLBCL, diffuse large B-cell lymphoma; DLBCL and seronegative RA, diffuse large B-cell lymphoma and seronegative rheumatoid arthritis; DLBCL and RA seropositive RA, diffuse large B-cell lymphoma and seropositive rheumatoid arthritis; IRD, inflammatory rheumatic disease; RA, rheumatoid arthritis.

**Figure 4:**
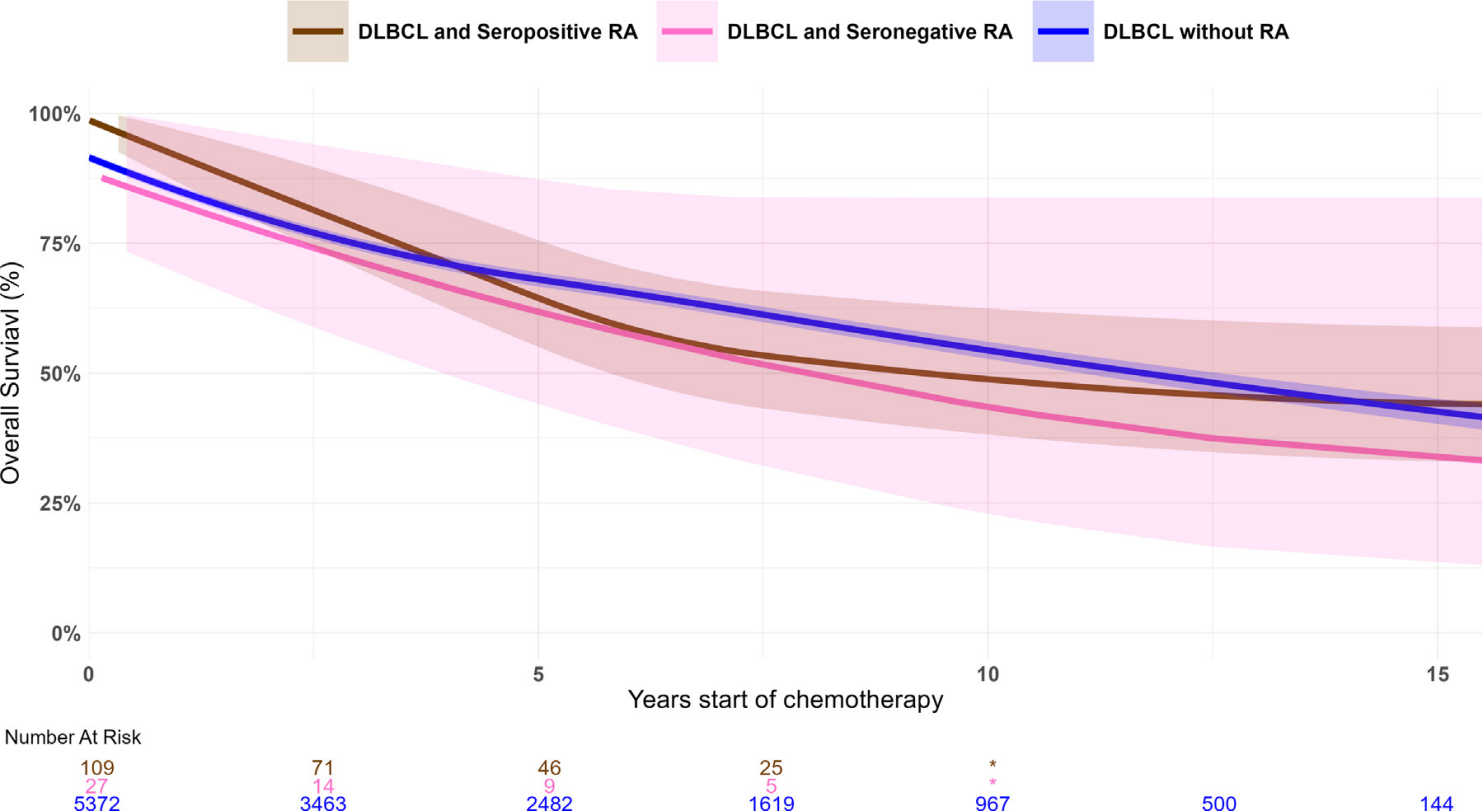
Smoothed Kaplan-Meier curve for overall survival in subgroup analysis comparing patients with DLBCL-RA with patients with DLBCL and RA treated with biological DMARDs and DLBCL patients with RA never treated with any form of biological DMARDs. *Number at risk censored due to Danish law on indirect anonymisation. DLBCL, diffuse large B-cell lymphoma; DLBCL and RA bio naïve, diffuse large B-cell lymphoma and rheumatoid arthritis never treated with any form of biological disease-modifying antirheumatic drugs; DLBCL and RA + bio, diffuse large B-cell lymphoma and rheumatoid arthritis previously treated with biological disease-modifying antirheumatic drugs; DLBCL-RA, diffuse large B-cell lymphoma and no inflammatory rheumatic disease; DMARD, disease-modifying antirheumatic drug; RA, rheumatoid arthritis.

**Figure 5:**
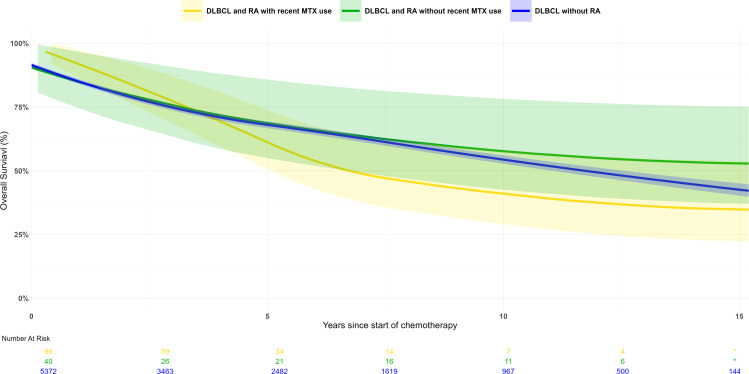
Smoothed Kaplan-Meier curve for overall survival in subgroup analysis, comparing patients with DLBCL without RA, patients with DLBCL and RA with recent MTX use, and patients with DLBCL and RA with no recent MTX use. *Number at risk censored due to Danish law on indirect anonymisation. DLBCL, diffuse large B-cell lymphoma; DLBCL and RA bio naïve, diffuse large B-cell lymphoma and rheumatoid arthritis never treated with any form of biological disease-modifying antirheumatic drugs; DLBCL and RA + bio, diffuse large B-cell lymphoma and rheumatoid arthritis previously treated with biological disease-modifying antirheumatic drugs; DLBCL without RA, diffuse large B-cell lymphoma and no inflammatory rheumatic disease; MTX, metrotrexate; RA, rheumatoid arthritis.

When stratifying patients with RA and DLBCL based on their history of bDMARD treatment, those who had received bDMARDs demonstrated a significantly higher 5-year OS compared with those who had never received bDMARDs (81.0% [95% CI, 60.1; 94.9] versus 54.4% [95% CI, 43.7; 67.7]). However, age at start of chemotherapy for patients in the bDMARD-treated group was 68.6 (IQR, 59.3 to 73.7) versus 73.2 (IQR, 68.1 to 78.5) in the bDMARD naïve group. After adjusting for various covariates, including age, the HR for death was 0.95 (95% CI, 0.52; 1.72). In both the subgroup analysis where patients were stratified by RA serostatus and the one where patients were stratified by recent methotrexate use, we found no notable differences in OS (Figs 3, 4, and 5) or HRs for death between the groups (Tables 3, 4, and 5).

**Table 3:** Subgroup analysis: overall survival, hazard of death comparing DLBCL and RA patients to patients with DLBCL and RA treated with biological DMARDs, and patients with DLBCL and RA never treated with any form of biological DMARDs

GroupNumber of patients	DLBCL and RA (+bDMARD) N = 42	DLBCL and RA (bDMARD naïve) N = 94	DLBCL without RA N = 5372
**Age at start of chemotherapy (IQR)**	68.6 [59.3, 73.7]	73.2 [68.1, 78.5]	68.6 [59.0, 76.1]
**Survival**			
5-y overall survival (95% CI)	80.9% (69.1; 94.8)	54.4% (43.7; 67.7)	68.9% (67.6; 70.2)
**Hazard ratios**			
Crude HR for death (95% CI)	0.70 (0.41; 1.21)	1.28 (0.93; 1.75)	Reference
Adjusted HR for death^a^ (95% CI)	0.95 (0.52; 1.72)	1.05 (0.72; 1.51)	Reference

DLBCL, diffuse large B-cell lymphoma; DLBCL and RA (+ bDMARD), diffuse large B-cell lymphoma and rheumatoid arthritis previously treated with biological disease-modifying antirheumatic drugs; DLBCL and RA (bDMARD naïve), diffuse large B-cell lymphoma and rheumatoid arthritis never treated with any form of biological disease-modifying antirheumatic drugs; DLBCL without RA, diffuse large B-cell lymphoma and no RA or any other inflammatory rheumatic disease; HR, hazard ratio; LDH, lactate dehydrogenase; RA, rheumatoid arthritis.

a Calculations adjusted for sex, age at lymphoma, performance status, Ann Arbor stage, extra nodal involvement, presence of B-symptoms, tumour bulk, and blood levels of LDH, 95% CI.

**Table 4:** Subgroup analysis of overall survival, hazard of death comparing DLBCL and RA patients to patients with DLBCL and seropositive RA, and DLBCL patients with seronegative RA

GroupNumber of patients	DLBCL and RA (seropositive) N = 109	DLBCL and RA (seronegative) N = 27	DLBCL without RA N = 5372
**Age at start of chemotherapy (IQR)**	71.9 [64.9, 78.2]	74.6 [68.8, 77.4]	68.6 [59.0, 76.1]
**Survival**			
5-y overall survival (95% CI)	64.4% (55.0; 75.4)	58.5% (40.1; 85.3)	68.9% (67.6; 70.2)
**Hazard ratios**			
Crude HR of death (95% CI)	1.02 (0.75; 1.39)	1.25 (0.69; 2.26)	Reference
Adjusted HR of death^a^ (95% CI)	1.06 (0.76; 1.49)	0.78 (0.32, 1.87)	Reference

DLBCL, diffuse large B-cell lymphoma; DLBCL and RA seropositive RA, diffuse large B-cell lymphoma and seropositive rheumatoid arthritis; DLBCL and seronegative RA, diffuse large B-cell lymphoma and seronegative rheumatoid arthritis; DLBCL without RA, diffuse large B-cell lymphoma and no RA or any other inflammatory rheumatic disease; HR, hazard ratio; LDH, lactate dehydrogenase; RA, rheumatoid arthritis.

a Calculations adjusted for sex, age at lymphoma, performance status, Ann Arbor stage, extra nodal involvement, presence of B-symptoms, tumour bulk, and blood levels of LDH, 95% CI.

**Table 5:** Subgroup analysis: overall survival, hazard of death comparing DLBCL without RA patients with DLBCL + RA patients with recent MTX use, and patients with DLBCL + RA and no recent MTX use

GroupNumber of patients	DLBCL and RA (recent MTX use) N = 96	DLBCL and RA (no recent MTX use) N = 40	DLBCL without RA N = 5372
**Age at start of chemotherapy (IQR)**	71.5 [59.3, 73.7]	70.2 [68.1, 78.5]	68.6 [59.0, 76.1]
**Survival**			
5-y overall survival (95% CI)	59.6% (49.1; 72.4)	69.6% (55.9; 82.5)	68.9% (67.6; 70.2)
**Hazard ratios**			
Crude HR for death (95% CI)	1.16 (0.84; 1.59)	0.89 (0.53; 1.47)	Reference
Adjusted HR for death^a^ (95% CI)	1.04 (0.72; 1.51)	0.96 (0.33, 1.74)	Reference

DLBCL and RA (no recent MTX use), diffuse large B-cell lymphoma and rheumatoid arthritis with no methotrexate within 6 months prior to the DLBCL diagnosis; DLBCL and RA (recent MTX use), diffuse large B-cell lymphoma and rheumatoid arthritis with methotrexate within 6 months prior to the DLBCL diagnosis; DLBCL + RA, diffuse large B-cell lymphoma and rheumatoid arthritis; DLBCL without RA, diffuse large B-cell lymphoma and no RA or any other inflammatory rheumatic disease; HR, hazard ratio; LDH, lactate dehydrogenase; MTX, metrotrexate.

a Calculations adjusted for sex, age at lymphoma, performance status, Ann Arbor stage, extra nodal involvement, presence of B-symptoms, tumour bulk, and blood levels of LDH, 95% CI.

## DISCUSSION

In this nationwide observational study of patients with DLBCL, we found no indications of worse clinical outcomes in DLBCL patients with RA compared with DLBCL without RA. DLBCL + RA patients did not experience lower OS nor PFS, and for hospital admissions specifically, a significantly lower risk was seen for DLBCL + RA patients compared with DLBCL without RA patients.

We found no significant differences in OS between DLBCL + RA and DLBCL without RA patients, with a corresponding HR for death of 1.01 (95% CI, 0.81; 1.39) with DLBCL without RA as the reference. These findings are corroborated by results from a study by Koff et al [16], which included 5926 elderly patients with DLBCL, of whom 155 had RA. This study found no significant difference in mortality for patients with DLBCL and RA compared with those without RA with an HR of 0.86 (95% CI, 0.69-1.08), and another study by Kleinstern et al [17] reported similar findings: HR of 1.15 (95% CI, 0.78-1.69). In contrast, a Swedish study by Ji et al [15], which included 329 patients with RA and 56,936 without RA, reported a significantly higher mortality in patients with NHL and RA compared with those with NHL without RA (HR, 1.33; 95% CI, 1.26; 1.59). However, this study considered all NHLs combined rather than restricting to DLBCL, and their study period went far back in time with DLBCL patients diagnosed during 1961-2006, which could explain the discrepancy between their findings and ours.

Interestingly, when looking at PFS, complete remission rates, and death 3 months after DLBCL diagnosis, DLBCL + RA patients showed signs of more favourable outcomes compared with DLBCL without RA patients. These tendencies were supported by the findings in a study from Mikuls et al [18], which reported that patients with DLBCL and RA tend to have better NHL-related outcomes compared with non-RA controls. The reason why patients with RA would experience more favourable lymphoma-related outcomes is unknown, but there have been documented case studies of spontaneous regression of lymphoma in individuals with RA following the discontinuation of immunosuppressive therapy, particularly methotrexate [[34], [35], [36], [37]]. Although spontaneous lymphoma regression has been documented, it is unlikely to have influenced our results, as all patients were evaluated by a specialist and deemed eligible to receive R-CHOP. Any DLBCL cases that regressed after methotrexate discontinuation would likely have done so prior to R-CHOP initiation and were therefore not included in the study.

Another consideration is the possibility that lymphomas, including DLBCL, arising in RA may differ biologically from those occurring in the general population. Studies have reported that DLBCL in RA patients more frequently exhibits a non-germinal centre subtype and Epstein–Barr virus positivity, both of which are typically associated with poorer outcomes [16,38]. Although these things may not explain the observed trends found in our study, they raise the possibility that RA-associated DLBCL may represent a biologically distinct entity rather than a conventional DLBCL occurring in a patient with RA.

In terms of hospitalisations, DLBCL + RA patients also had a lower IR for the number of inpatient bed days compared with DLBCL without RA patients, with 159 and 245 inpatient bed days per 100 PYs, respectively. The corresponding finding for first-time hospital admission for DLBCL + RA patients was HR 0.56 (95% CI, 0.44; 0.72). Perhaps these fewer hospitalisations among the DLBCL + RA patients could be attributed to the nature of the care of patients with RA in general, as they are often monitored closely by outpatient clinics and primary care physicians—possibly leading to a more proactive management of health issues outside of an inpatient hospital setting. For our sensitivity analysis, where we excluded patients admitted due to the administration of chemotherapy, and for the subgroup analysis, where we stratified RA patients based on seropositivity, history of treatment with bDMARD, and prior methotrexate use, we did not find any significant difference in results.

Our study has some limitations. We chose not to adjust for specific comorbidities at baseline, eg, cardiovascular disease, kidney disease, and lung disease [[39], [40], [41]]. This decision was based on views of RA as a systemic inflammatory disease, which in itself might cause these ‘comorbidities’ in the first place, and potentially adjusting for them would be adjusting for some of the underlying RA disease’s impact on the clinical cancer outcomes. However, there might be differences in comorbidities beyond what can be explained by the underlying RA, thereby leading to residual confounding. Another potential limitation lies in our reliance on DNPR to identify IRD cases within the comparator group. The DNPR only captures diagnoses made in hospital settings, meaning individuals diagnosed or managed solely in primary care or private clinics are not represented. Moreover, a single recorded diagnosis of an IRD in the DNPR would result in exclusion from the comparator group. As such, a single misclassification due to diagnostic errors or one-time incorrect entries would exclude a patient from the comparator group. Additionally, patients with RA may be diagnosed with DLBCL earlier due to more frequent hospital and primary care visits. This could explain the higher complete remission rate and lower HR for hospital admission. However, when examining clinicopathological features, RA + DLBCL patients had a higher Ann Arbor stage at diagnosis compared with DLBCL without RA patients, which usually indicates a more advanced disease. To account for differences in clinicopathological features, we adjusted for many of the known prognostic factors related to DLBCL, but there may be other relevant prognostic factors that we were unable to measure and adjust for. Lastly, due to the inclusion of only 136 patients with DLBCL and RA, the study may lack sufficient power to detect potentially important differences between the groups, and because the expected effect sizes for most of the outcomes in our cohorts were largely unknown, any formal power calculation would be mostly speculative. Instead, we chose to focus on methodological rigour in order to provide as specific and unbiased effect estimates as possible [42].

Strengths of the study include the linkage of nationwide registers DANBIO and LYFO with high completeness and PPVs >95% of DLBCL and RA diagnoses. In addition to OS, we were able to investigate various other clinically relevant DLBCL outcomes registered in LYFO with a few missing values. Furthermore, using LYFO, we were also able to adjust for important potential DLBCL-specific confounders. Lastly, this is to our knowledge the first study to specifically compare PFS, remission rates, and hospital admissions between DLBCL + RA patients and those without RA in contrast to previous studies investigating mortality only.

In conclusion, our registry-based study of Danish DLBCL patients did not find worse clinical outcomes for those with RA compared with those without RA; no differences in the risk of death, PFS, and serious infections were found, and the risk of hospital admission and number of inpatient bed days even favoured DLBCL patients with RA. However, additional studies on the outcomes of patients with DLBCL and RA are warranted to better understand and potentially further improve the treatment of this group of DLBCL patients.

## CRediT authorship contribution statement

**Bergur Magnussen:** Writing – review & editing, Writing – original draft, Visualization, Validation, Supervision, Software, Resources, Project administration, Methodology, Investigation, Funding acquisition, Formal analysis, Data curation, Conceptualization. **Lene Wohlfart Dreyer:** Writing – review & editing, Validation, Supervision, Resources, Project administration, Methodology, Investigation, Funding acquisition, Conceptualization. **Salome Kristensen:** Writing – review & editing, Validation, Supervision, Resources, Project administration, Methodology, Investigation, Funding acquisition, Conceptualization. **Tarec Christoffer El-Galaly:** Writing – review & editing, Validation, Supervision, Resources, Methodology, Investigation, Data curation, Conceptualization. **Lasse Hjort Jakobsen:** Writing – review & editing, Visualization, Software, Methodology, Data curation, Conceptualization. **Lene Mellemkjær:** Writing – review & editing, Validation, Supervision, Methodology, Funding acquisition, Conceptualization. **Mikkel Simonsen:** Writing – review & editing, Software, Methodology. **Peter Brown:** Writing – review & editing, Validation, Methodology. **Rasmus Westermann:** Writing – review & editing, Visualization, Validation, Supervision, Software, Resources, Project administration, Methodology, Investigation, Funding acquisition, Formal analysis, Data curation, Conceptualization.

## Appendix Supplementary materials

Image, application 1

## Footnotes

Supplementary material associated with this article can be found in the online version at doi:10.1016/j.ero.2025.11.009.

## References

[1] Thandra K.C., Barsouk A., Saginala K., Padala S.A., Barsouk A., Rawla P. (2021). Epidemiology of non-Hodgkin’s lymphoma. Med Sci (Basel).

[2] Westermann R., Zobbe K., Cordtz R., Haugaard J.H., Dreyer L. (2021). Increased cancer risk in patients with cutaneous lupus erythematosus and systemic lupus erythematosus compared with the general population: a Danish nationwide cohort study. Lupus.

[3] Cordtz R.L., Askling J., Delcoigne B., Smedby K.E., Baecklund E., Ballegaard C. (2022). Haematological malignancies in patients with psoriatic arthritis overall and treated with TNF inhibitors: a Nordic cohort study. RMD Open.

[4] Anderson L.A., Gadalla S., Morton L.M., Landgren O., Pfeiffer R., Warren J.L. (2009). Population-based study of autoimmune conditions and the risk of specific lymphoid malignancies. Int J Cancer.

[5] Ekström Smedby K., Vajdic C.M., Falster M., Engels E.A., Martínez-Maza O., Turner J. (2008). Autoimmune disorders and risk of non-Hodgkin lymphoma subtypes: a pooled analysis within the InterLymph Consortium. Blood.

[6] Baecklund E., Sundström C., Ekbom A., Catrina A.I., Biberfeld P., Feltelius N. (2003). Lymphoma subtypes in patients with rheumatoid arthritis: increased proportion of diffuse large B cell lymphoma. Arthritis Rheum.

[7] Hellgren K., Di Giuseppe D., Smedby K.E., Sundström C., Askling J., Baecklund E. (2021). Lymphoma risks in patients with rheumatoid arthritis treated with biological drugs-a Swedish cohort study of risks by time, drug and lymphoma subtype. Rheumatology (Oxford).

[8] Hellgren K., Baecklund E., Backlin C., Sundstrom C., Smedby K.E., Askling J. (2017). Rheumatoid arthritis and risk of malignant lymphoma: is the risk still increased?. Arthritis Rheumatol.

[9] Klein A., Polliack A., Gafter-Gvili A. (2018). Rheumatoid arthritis and lymphoma: incidence, pathogenesis, biology, and outcome. Hematol Oncol.

[10] Simon T.A., Thompson A., Gandhi K.K., Hochberg M.C., Suissa S. (2015). Incidence of malignancy in adult patients with rheumatoid arthritis: a meta-analysis. Arthritis Res Ther.

[11] Baecklund E., Iliadou A., Askling J., Ekbom A., Backlin C., Granath F. (2006). Association of chronic inflammation, not its treatment, with increased lymphoma risk in rheumatoid arthritis. Arthritis Rheum.

[12] Mariette X., Cazals-Hatem D., Warszawki J., Liote F., Balandraud N., Sibilia J. (2002). Lymphomas in rheumatoid arthritis patients treated with methotrexate: a 3-year prospective study in France. Blood.

[13] Kamel O.W., Van De Rijn M., Lebrun D.P., Weiss L.M., Warnke R.A., Dorfman RF. (1994). Lymphoid neoplasms in patients with rheumatoid arthritis and dermatomyositis: frequency of Epstein-Barr virus and other features associated with immunosuppression. Hum Pathol.

[14] Liang X., Hu R., Li Q., Wang C., Liu Y. (2023). Prognostic factors for diffuse large B-cell lymphoma: clinical and biological factors in the rituximab era. Exp Hematol.

[15] Ji J., Liu X., Sundquist K., Sundquist J. (2011). Survival of cancer in patients with rheumatoid arthritis: a follow-up study in Sweden of patients hospitalized with rheumatoid arthritis 1 year before diagnosis of cancer. Rheumatology (Oxford).

[16] Koff J.L., Rai A., Flowers CR. (2018). Characterizing autoimmune disease-associated diffuse large B-cell lymphoma in a SEER–Medicare cohort. Clin Lymphoma Myeloma Leuk.

[17] Kleinstern G., Averbuch M., Abu Seir R., Perlman R., Ben Yehuda D., Paltiel O. (2018). Presence of autoimmune disease affects not only risk but also survival in patients with B-cell non-Hodgkin lymphoma. Hematol Oncol.

[18] Mikuls T.R., Endo J.O., Puumala S.E., Aoun P.A., Black N.A., O’Dell J.R. (2006). Prospective study of survival outcomes in non-Hodgkin’s lymphoma patients with rheumatoid arthritis. J Clin Oncol.

[19] Schmidt M., Pedersen L., Sørensen HT. (2014). The Danish Civil Registration System as a tool in epidemiology. Eur J Epidemiol.

[20] Pedersen CB. (2011). The Danish civil registration system. Scand J Public Health.

[21] Arboe B., Josefsson P., Jørgensen J., Haaber J., Jensen P., Poulsen C. (2016). Danish national lymphoma registry. Clin Epidemiol.

[22] Arboe B., El-Galaly T.C., Clausen M.R., Munksgaard P.S., Stoltenberg D., Nygaard M.K. (2016). The Danish National Lymphoma Registry: coverage and data quality. PLoS One.

[23] Bjerregaard B., Larsen OB. (2011). The Danish pathology register. Scand J Public Health.

[24] Hetland ML. (2005). DANBIO: a nationwide registry of biological therapies in Denmark. Clin Exp Rheumatol.

[25] Ibfelt E.H., Jensen D.V., Hetland ML. (2016). The Danish nationwide clinical register for patients with rheumatoid arthritis: DANBIO. Clin Epidemiol.

[26] Ibfelt E.H., Sørensen J., Jensen D.V., Dreyer L., Schiøttz-Christensen B., Thygesen P.H. (2017). Validity and completeness of rheumatoid arthritis diagnoses in the nationwide DANBIO clinical register and the Danish National Patient Registry. Clin Epidemiol.

[27] Schmidt M., Schmidt S.A.J., Sandegaard J.L., Ehrenstein V., Pedersen L., Sørensen HT. (2015). The Danish National Patient Registry: a review of content, data quality, and research potential. Clin Epidemiol.

[28] Lynge E., Sandegaard J.L., Rebolj M. (2011). The Danish national patient register. Scand J Public Health.

[29] Blichert-Hansen L., Nielsson M.S., Nielsen R.B., Christiansen C.F., Nørgaard M. (2013). Validity of the coding for intensive care admission, mechanical ventilation, and acute dialysis in the Danish national patient registry: a short report. Clin Epidemiol.

[30] Lawless J.F., Crowder MJ. (2010). Models and estimation for systems with recurrent events and usage processes. Lifetime Data Anal.

[31] Katchamart W., Koolvisoot A., Aromdee E., Chiowchanwesawakit P., Muengchan C. (2015). Associations of rheumatoid factor and anti-citrullinated peptide antibody with disease progression and treatment outcomes in patients with rheumatoid arthritis. Rheumatol Int.

[32] Carbonell-Bobadilla N., Soto-Fajardo C., Amezcua-Guerra L.M., Batres-Marroquín A.B., Vargas T., Hernández-Diazcouder A. (2022). Patients with seronegative rheumatoid arthritis have a different phenotype than seropositive patients: a clinical and ultrasound study. Front Med (Lausanne).

[33] Lauritzen F.F., Soussi B.G., Duch K.S., Kristensen S., Mortensen A.S., Dreyer L. (2024). OP0162 The temporal mortality trend in seropositive and seronegative rheumatoid arthritis - a Danish population-based cohort study. Ann Rheum Dis.

[34] Salloum E., Cooper D.L., Howe G., Lacy J., Tallini G., Crouch J. (1996). Spontaneous regression of lymphoproliferative disorders in patients treated with methotrexate for rheumatoid arthritis and other rheumatic diseases. J Clin Oncol.

[35] Georgescu L., Quinn G.C., Schwartzman S., Paget SA. (1997). Lymphoma in patients with rheumatoid arthritis: association with the disease state or methotrexate treatment. Semin Arthritis Rheum.

[36] Kameda T., Izumikawa M., Inoo M., Onishi I., Kurata N., Nakashima S. (2017). THU0209 Lymphocyte subsets in biopsy specimen are associated with spontaneous regression of lymphoproliferative disorders in rheumatoid arthritis patients treated with methotrexate. Ann Rheum Dis.

[37] Usman A.R., Yunus MB. (1996). Non-Hodgkin’s lymphoma in patients with rheumatoid arthritis treated with low dose methotrexate. J Rheumatol.

[38] Baecklund E., Backlin C., Iliadou A., Granath F., Ekbom A., Amini R.M. (2006). Characteristics of diffuse large B cell lymphomas in rheumatoid arthritis. Arthritis Rheum.

[39] Couderc M., Tatar Z., Pereira B., Tiple A., Gilson M., Fautrel B. (2016). Prevalence of renal impairment in patients with rheumatoid arthritis: results from a cross-sectional multicenter study. Arthritis Care Res (Hoboken).

[40] Mori S., Yoshitama T., Hirakata N., Ueki Y. (2017). Prevalence of and factors associated with renal dysfunction in rheumatoid arthritis patients: a cross-sectional study in community hospitals. Clin Rheumatol.

[41] Hill J., Harrison J., Christian D., Reed J., Clegg A., Duffield S.J. (2022). Central Lancashire Online Knowledge (CLoK) The prevalence of comorbidity in rheumatoid arthritis: a systematic review and meta-analysis: a systematic review. Br J Community Nurs.

[42] Hernán MA. (2022). Causal analyses of existing databases: no power calculations required. J Clin Epidemiol.

