# Supplementary material for: Clinical outcomes of patients with diffuse large B-cell lymphoma and concomitant rheumatoid arthritis: a nationwide Danish register-based cohort study
Source: EULAR Rheumatol Open. 2025 Dec 6;1(4):452–61. doi: 10.1016/j.ero.2025.11.009 (PMC13292481; doi:10.1016/j.ero.2025.11.009)
Supplement: Supplementary file 1 [file mmc1.docx]

**Supplementary material 1 list of excluded inflammatory rheumatic diseases.**

| **ICD-10 Code** | **Disease** |
| --- | --- |
| **M05** | **Rheumatoid arthritis with rheumatoid factor** |
| **M06** | **Other Rheumatoid artritis** |
| **M30** | **Polyarteritis nodosa and related conditions** |
| **M31** | **Other necrotizing vasculopathies** |
| **M32** | **Systemic lupus erythematosus** |
| **M33** | **Other, dermatomyositis organ involvement unspecified** |
| **M070** | **Distal interphalangeal psoriatic arthropathy** |
| **M071** | **Arthritis mutilans** |
| **M072** | **Psoriatic spondylitis** |
| **M073** | **Other psoratic arthropathies** |
| **M350** | **Sjögrens syndrom** |
| **M353** | **Polymyalgia rheumatica** |
| **M459** | **Ankylosing spondylitis of unspecified sites in spine** |
| **M460** | **Spinal enthesopathy** |
| **M461** | **Sacrolitis, not elsewhere classified** |
| **M469** | **Unspecified inflammatory spondylopathy** |
| **DL405** | **Psoriatic arthritis** |

**Supplementary materiel 2: list of ICD-10 codes included for hospitalisations related to infections.**

| **A00-A32, A34-A99, B003, B004, B01B99, D73.3, E32.1G00, G02, G04.2, G06, G07**  **H00.0,H01.0,H03.1,H06.1,H10,H13,H16,H19.1,H19.2,H22,H30,H44.0,H60.0,H60.1,H60.2,H60.3,H62,H66,H67,H70,H73,H75.0,H94.0,I30.1,I33.0,I39,I140.0,I152,I168.1,I198, J00-J22, J32, J34.0, J36, J38.3, J39.0-J39.1, J44.0, J85, J86, K04.4, K04.6, K04.7, K10.2, K11.3, K12.2, K14.0, K57.0, K57.2, K57.4, K57.8, K61, K63.0, K65.0, K65.1, K65.2, K65.9, L00-L08, L30.3, M00-M01, M46.2-M46.5, M60.0, M65.0, M71.0, M71.1, M72.6, M86, N10, N11, N12, N13.6, N15.1, N15.9, N30.0 N30.8, N34.0, N41.2, N43.1, N45.2, N45.3, N45.4,N48.2,N61,N70,N73,N75.1,N76.0,N77.0,N77.1,O07,O08.0,O23,O26.4,O35.3,O41.1,O75.3,O85,O86,O88.3,O91,O98** |
| --- |
